# Proteome driven re-evaluation and functional annotation of the *Streptococcus pyogenes *SF370 genome

**DOI:** 10.1186/1471-2180-11-249

**Published:** 2011-11-10

**Authors:** Akira Okamoto, Keiko Yamada

**Affiliations:** 1 Department of Molecular Bacteriology, Nagoya University Graduate School of Medicine, 65 Tsurumai-cho, Showa-ku, Nagoya, Aichi 466-8550, Japan

## Abstract

**Background:**

The genome data of *Streptococcus pyogenes *SF370 has been widely used by many researchers and provides a vast array of interesting findings. Nevertheless, approximately 40% of genes remain classified as hypothetical proteins, and several coding sequences (CDSs) have been unrecognized. In this study, we attempted a shotgun proteomic analysis with a six-frame database that was independent of genome annotation.

**Results:**

Nine proteins encoded by novel ORFs were found by shotgun proteomic analysis, and their specific mRNAs were verified by reverse transcriptional PCR (RT-PCR). We also provided functional annotations for hypothetical genes using proteomic analysis from three different culture conditions that were separated into three fractions: supernatant, soluble, and insoluble. Consequently, we identified 567 proteins on re-evaluation of the proteomic data using an in-house database comprising 1,697 annotated and nine non-annotated CDSs. We provided functional annotations for 126 hypothetical proteins (18.9% out of the 668 hypothetical proteins) based on their cellular fractions and expression profiles under different culture conditions.

**Conclusions:**

The list of amino acid sequences that were annotated by genome analysis contains outdated information and unrecognized protein-coding sequences. We suggest that the six-frame database derived from actual DNA sequences be used for reliable proteomic analysis. In addition, the experimental evidence from functional proteomic analysis is useful for the re-evaluation of previously sequenced genomes.

## Background

Comprehensive molecular biological approaches, including genome, transcriptome, proteome, and metabolome analyses are powerful, essential tools for understanding the phenotype of all living organisms. In recent years, high-throughput DNA sequencing technologies have enabled the sequencing of a microbial genome in a few days. However, the identification, annotation, and curation of genes have been limiting factors in the analysis of new genomes. The criteria for identifying and annotating genes depend on the curator. Usually, curators should annotate all open reading frames (ORFs) based on the features of promoter regions, such as the presence or absence of Shine-Dalgarno sequences, and based on homology searches with nucleic acid databases. Moreover, databases such as NCBInr in the National Center of Biotechnology Information (NCBI) have been updated, although microbial genomes seem to contain several "conserved hypothetical protein (CHyP)" or "hypothetical protein (HyP)", and unrecognized coding sequences (CDSs) [1]. The revision of previously published genomes is a concern for many researchers; however, there are only a few cases of revisions of original genome annotations in public databases [2-4]. Several studies reported the evaluation of published genomes by developed ORF finding algorithms with expended databases [5-8]. Another approach for genome re-evaluation was performed using support from experimental evidence, such as transcriptomic or proteomic analysis [4,8-13].

*Streptococcus pyogenes*, group A streptococci (GAS) is an important human pathogen that causes various infectious diseases, including pharyngitis, scarlet fever, impetigo, necrotizing fasciitis, and streptococcal toxic shock-like syndrome. Efforts have been made to illustrate the proteomic profile of GAS, as several secreted or membrane-associated proteins from this pathogen are responsible for these diseases [14-16]. GAS SF370 is a significant strain that has been widely used in research because its genome has been available since 2001[17]. Since then, another 12 GAS genomes have become available [18-25]. However, approximately 40% of SF370 genes still remained annotated as CHyP or HyP. Furthermore, the number of annotations has approximately 100 fewer protein-coding sequences (CDSs) compared to other sequenced GAS strains that possess almost the same genome, both in terms of composition and size [26]. It is assumed that a number of unrecognized CDSs reside in the relatively larger intergenic regions or overlap another reading frame. In fact, we previously identified two proteins that we deduced to be encoded by unrecognized CDS in SF370 [27].

In the present study, we attempted to identify unrecognized CDSs in SF370 and verified the mRNA expressions of these CDSs using reverse transcription PCR (RT-PCR). In addition, proteomic analysis provided functional annotations for CHyPs and HyPs in SF370. The revision of the annotation should provide useful information for researchers studying this pathogen.

## Results

### Intra-species Genomic Overview of GAS

The genomes of 13 *S. pyogenes *strains have been sequenced, and the number of protein coding genes that have been annotated in each genome ranged from 1,696 (SF370) to 1,987 (MGAS10270). The total length of the MGAS10270 genome was 78,812 bp greater than that of SF370, and contains 100 more CDSs than that of SF370. To summarize the variations in genome analysis data of *S. pyogenes*, each genome feature is listed in Additional file 1. CDS coverage was estimated from the total length of CDSs that were annotated in each genome. The average genome length of the 13 strains of *S. pyogenes *was 1,864,731 bp, the average CDS coverage was 88.11%, the average number of genes was 1,941, the average length of protein coding genes was 872 bp, and the average number of protein coding genes was 1,855. SF370 was the first GAS strain to be sequenced in 2001 and it had a comparatively lower CDS coverage (86.94%) and fewer number of protein coding genes (1,696) than other GAS strains. In contrast, its average length of protein coding genes (915 bp) was the highest. Although the genome of MGAS5005 serotype M1 exhibited differences in several of its prophage contents, small insertions or deletions, and SNPs, its gene components were similar to that of SF370 [26]. The number of protein coding genes annotated for MGAS5005 chromosome was 197 more than that for SF370, whereas the chromosome size of MGAS5005 was 13,886 bp greater than that of SF370. This difference in total genome length should correspond to 15-16 protein-coding genes based on the average length of protein coding genes. These results indicated that several genes might have been unrecognized among the CDSs in SF370.

### Expression of Unrecognized CDSs in SF370

A mixture of the tryptic-digested proteins of SF370 was applied to liquid chromatography combined with tandem mass spectrometry (LC-MS/MS). The digested products were separated using a reversed linear gradient. An overview of the shotgun proteomic analysis is shown in Additional file 2. To find unrecognized CDSs in SF370 genome annotation, the product ion mass lists were queried using the MASCOT program and an in-house database comprising 197,566 six-frame ORFs. A total of 487 ORFs were identified through all LC-MS/MS shotgun experiment. The number of ORFs that corresponded to known CDS was 478, and nine ORFs were found to be CDS candidates that were unrecognized in the SF370 genome annotation (Additional file 3).

BLASTP searches revealed that these nine CDS candidates shared high homology (E values 0.0 - 2 × 10^-54^) with genes that were annotated in other GAS genome analyses. These nine new CDSs were further annotated by sequence homology searches in the Gene Ontology (GO) database. All the CDS, except for ORF6306, were assigned with GO terms. Three out of the nine new ORFs were assigned to "cellular component" GO terms, which largely agreed with the experimental evidence from the proteomic analysis (Additional file 3).

Oligopeptide permease periplasmic binding protein (OppA; ORF 13562) and two component response regulators, CsrR, (ORF15403) were previously found in the SF370 supernatants [27]. ORF125651 shares homology with peptidyl-prolyl cis-trans isomerase, which was annotated with tagged M5005_Spy_1331 in the MGAS5005 genome (EC 5.2.1.8). GO annotation indicated that the product of ORF125651 is involved in protein folding. ORF6306 shared homology with fibronectin-binding protein, which was annotated with tagged M5005_Spy_0107 in the MGAS5005 genome. Although ORF6306 was not assigned any GO terms, it was estimated to possess two membrane-spanning domains by the SOSUI program, and a signal sequence by the SignalP program. These primary structure-based features seemed to be reasonable because the peptides assigned to ORF6306 were mainly detected in the insoluble fraction under all culture conditions [28-30]. Taken together, the results suggest that the product encoded by ORF6306 is located near the outer side of the cell, probably in the cell wall. ORF703 is homologous to a small protein with a molecular weight of 20,594, hypoxanthine-guanine phosphoribosyltransferase, which was annotated in the MGAS8232 genome. ORF3228 showed homology with a bifunctional acetaldehyde-CoA/alcohol dehydrogenase (Adh2, EC numbers of 1.2.1.10 and 1.1.1.1), which was annotated with tagged M5005_SPy_0039 in the MGAS5005 genome. Relatively large numbers of peptide sequences (12 - 23) were detected in the soluble and insoluble fractions under static and CO_2 _culture conditions, whereas no peptides were identified in shaking condition. ORF123848 shared homology with thioredoxin reductase, which was annotated with tagged M5005_Spy_1360 in the MGAS5005 genome. The product of ORF123848 estimated to be involved in oxidation reduction by GO annotation. ORF5890 shared homology with a relatively small molecular weight (22,439) tRNA-binding domain-containing protein, which was annotated with tagged M5005_Spy_0101 in the MGAS5005 genome. ORF106976 shared homology with a relatively small molecular weight (11,354) hypothetical protein in MGAS315 tagged with SpyM3_1741. This small protein shared homology with part of the pyrogenic exotoxin B (SpeB); however, the peptide fragments assigned to ORF106976 in this study showed no identity with the amino acid sequence of SpeB (data not shown).

In summary, proteomic-assisted re-annotation of the SF370 genome with an in-house database consist of six-frame ORFs identified novel nine ORFs as candidate CDSs that are expressed in SF370.

### Detection of mRNAs of Novel CDS Candidates

**Figure 1:**
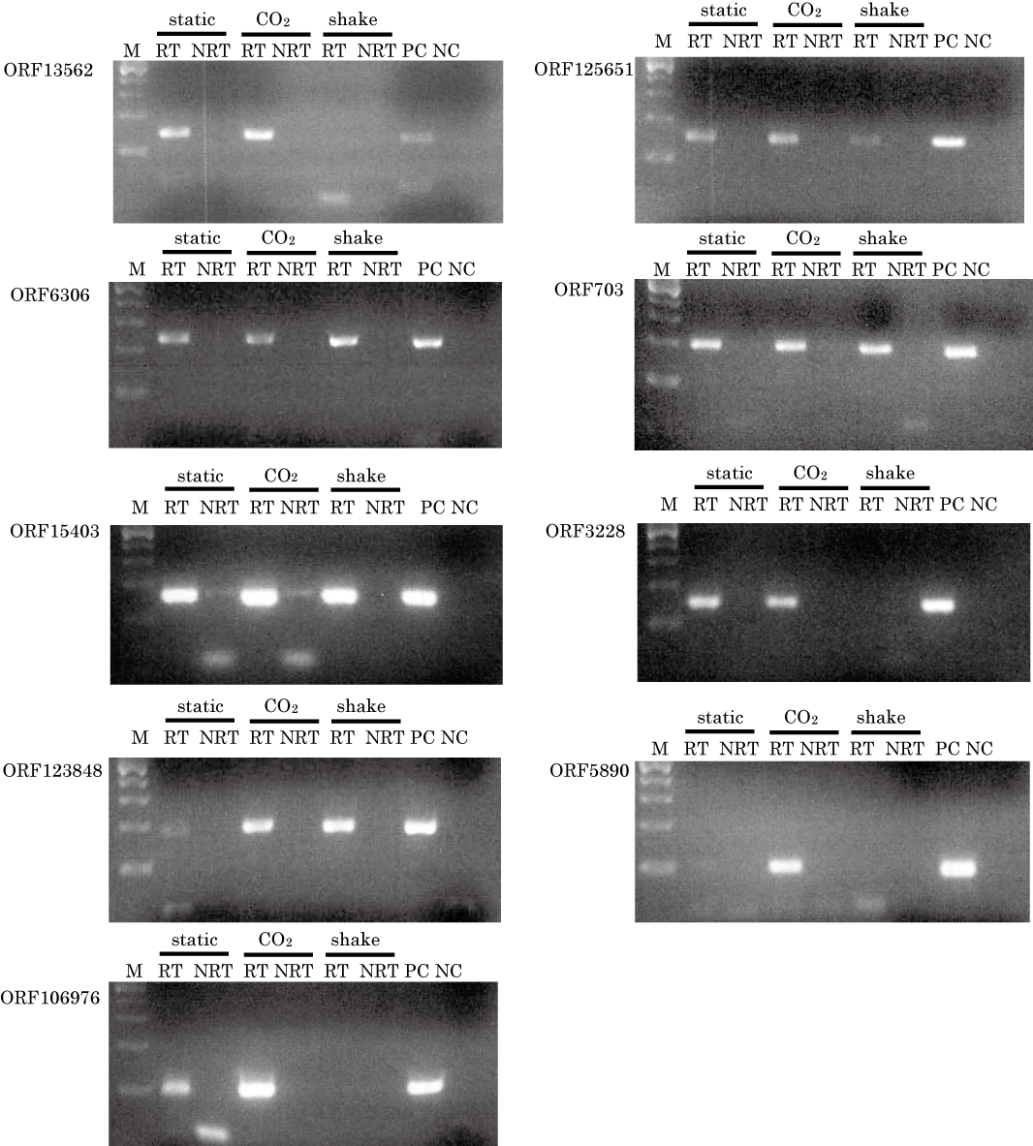
**RT-PCR confirmation of candidate ORFs**. mRNAs corresponding to candidate ORFs were evaluated by RT-PCR (RT). In both cases, RT-PCR used no transcriptase-containing sample (NRT) and PCR with no template (NC) as negative controls and PCR with genomic DNA as a positive control (PC).

RT-PCR analysis of candidate CDSs was used to verify the transcription of the mRNAs of these genes. The results of RT-PCR were consistent with the shotgun proteomic analysis. RT-PCR amplified the mRNAs of all nine candidate CDSs, verifying the transcription of these genes (Figure 1, Additional file 3). Although some mRNAs, corresponding to ORF13562 and ORF5890 under shaking conditions, were not detected by RT-PCR analysis, almost the entire mRNA expression pattern was in agreement with the proteomic analysis. To amplify the mRNAs derived from ORF13562 and ORF5890 under shaking conditions, we increased the number of RT-PCR cycles from 30 to 40. However, the amplified PCR products obtained by reverse transcription of total RNA samples were similar to those from the mock (non-reverse transcription) control. In shaking culture condition, these mRNAs may be expressed at a level that is below the detection threshold of the RT-PCR conditions used.

### Comparative Proteomic Analysis for Different Culture Conditions

Shotgun LC-MS/MS proteomic analysis revealed the expressions of 567 proteins out of 1,706 CDSs (nine novel CDSs with 1,697 CDSs in the genome annotation) under three differential culture conditions, including under atmospheric conditions with or without shaking, and under 5% CO_2 _(Additional file 4 Figure 2). Of these 567 proteins, 328 proteins (57.8%) were commonly identified under all culture conditions; 105 proteins (18.5%) were identified under more than two culture conditions, and the remaining 134 proteins (23.6%) were identified only under one culture condition each. In the supernatant, soluble fraction, and insoluble fraction, the number of proteins commonly identified under three different culture conditions were 33 (30.8%), 273 (58.7%), and 235 (53.3%), respectively. This result indicated that these commonly identified proteins comprised a core set of SF370 proteins, at least during the stationary phase. These results also suggested that variations in secreted proteins were more likely than for cell body-associated proteins as SF370 cells adapted to the environmental conditions.

**Figure 2:**
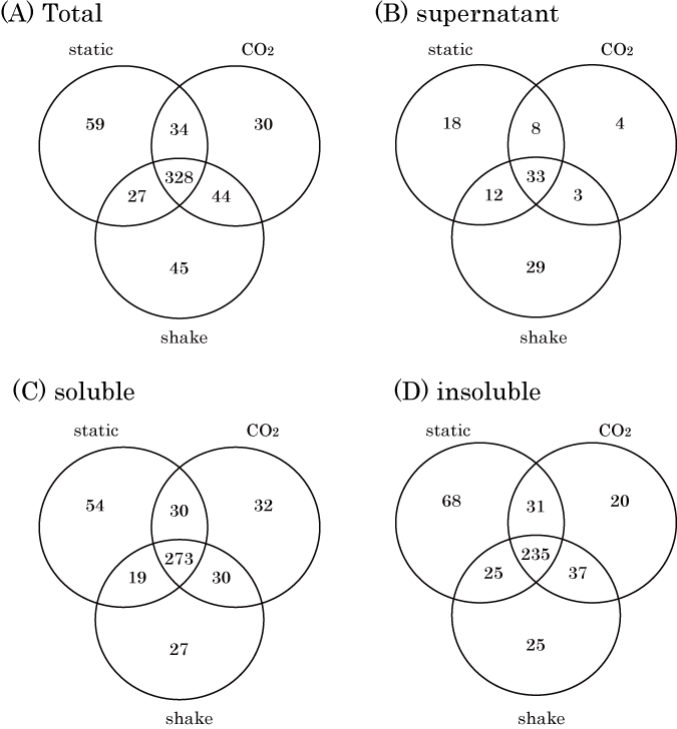
**Venn diagram of the distributions of identified proteins under each culture condition**. The distribution of total identified proteins under each culture condition is indicated (A). Numbers of proteins in the supernatant (B), soluble fraction (C), and insoluble fraction (D) are also shown.

### Functional Annotations for Hypothetical Proteins

The proportion of "conserved hypothetical protein (CHyP)" or "hypothetical protein (HyP)" accounts for 39.4% (346 genes for CHyP and 322 genes for HyP) of all annotated genes in the SF370 genome. We assigned functional annotations to these CHyP or HyP genes with LC-MS/MS shotgun proteomic analysis. In this study, we identified the products of 84 CHyP (24.3% of all CHyP) and 42 HyP (13.0% of all HyP) genes, respectively (Additional file 5 and 6). To update the annotations for these hypothetical genes, we divided these CHyP and HyP genes into expression pattern groups based on the cell fraction and culture conditions. We assumed that the cellular fraction would reflect a protein's location in bacterial culture. For example, a protein that was identified only in the supernatant should be categorized into the secreted protein group, or a protein that was identified in the soluble and insoluble fractions, but not in the supernatant, should be categorized in the whole cell-associated group. More than twice the number of assigned unique peptide sequences was used for these criteria to estimate the protein expression pattern. These 126 hypothetical proteins were classified on the basis of their cellular locations as follows: 41 cytoplasmic proteins, 34 cell wall-associated proteins, 10 secreted proteins, 35 whole cell-associated proteins, two cytoplasmic and secreted proteins, and four universally located proteins. SPy0747, which was estimated to possess two membrane spanning domains and a relatively high signal peptide score (0.877 in HMM prediction), showed a tendency to be located near the outer side of the cell, rather than in the cytoplasmic fraction. The expression profiles based on culture conditions were also similarly classified into groups. Twenty-five proteins were expressed only under static conditions. Thirteen proteins were expressed only under 5% CO_2 _conditions. Twenty proteins were expressed only under shaking conditions. Ten proteins were expressed under both static and CO_2 _conditions. Seven proteins were expressed under both static and shaking conditions. Fifteen proteins were expressed under CO_2 _and shaking conditions, and 36 proteins were expressed under all three culture conditions. The product encoded by SPy0792, which was identified in the insoluble fraction under atmospheric culture conditions with or without shaking, was consistent with the annotation for a CHyP that was "possibly involved in cell wall localization and side chain formation of rhamnose-glucose polysaccharide". Three hypothetical proteins, SPy0697, SPy0702, and SPy0998, were identified under static culture conditions. These three proteins were included in a specific prophage region associated with SF370 and its related strains [31]. SPy0697 and SPy0702 were included in φSP370.1, and the virulence factors *speC *and *mf2 *were encoded in this prophage region. SPy0998 was included in φSF370.2, and the virulence factors *speI *and *speH *were encoded in this prophage region.

To extensively annotate these hypothetical proteins, GO terms, estimation for membrane spanning domains (SOSUI), and signal sequence for secretion (SignalP) were integrated (Additional file 5 and 6). Three classes of GO terms, cellular component, biological process, and molecular function were assigned to 79 hypothetical proteins; however, 47 proteins could not be linked to any GO terms.

## Discussion

Comprehensive molecular biological approaches, such as transcriptome or proteome analysis, are essential for understanding the phenomenon of infection caused by virulent organisms, including GAS. Most post-genomic analysis is undertaken based on annotations derived from genome research. However, as mentioned above, previous genome analysis identified a number of "hypothetical proteins" that possibly represent unrecognized CDSs. Typical genome analysis is performed using a search procedure based on similarities. A query sequence derived from a list of ORFs in a genome is searched against a database comprising known amino acid sequences. These databases, such as NCBInr, have increased in size exponentially. Several genomes were re-evaluated semi-automatically with developed programs for gene identification [3,5-7]. In an intra-species genomic overview of *S. pyogenes*, gene prediction was largely divided into two groups depending on whether the gene predictor ERGO was used or not (Additional file 1) [32-35]. Genes were predicted by ERGO in seven out of 13 *S. pyogenes *genome analyses, with an average CDS coverage 89.05% in the genome and an average length of protein coding gene of 861 bp. On the other hand, other gene prediction programs were used in the other five analyses, generating an average CDS coverage of 86.61% in genome, and an average length of protein coding genes of 890 bp. This suggested that the ERGO system predicted shorter ORFs compared to other gene predictors. It could be that the ERGO system over-predicted genes, whereas these genes might have been dismissed by the other gene predictors. The issue of trade-off between unrecognized ORF and over-prediction of genes should be solved using experimental evidence.

In fact, methods for gene prediction have been developed, and novel CDSs have been found by experimentally supported approaches [2,8,13]. Dandekar et al. revised the *Mycoplasma pneumoniae *genome and increased the total number of ORFs from 677 to 688 by integration of a gene-identifying program and proteomic experiments [2]. They found 10 new CDSs in intergenic regions, two were identified by 2-dimensional gel electrophoresis followed by mass spectrometry, and one ORF was dismissed. The public genome annotation (GenBank: U00089) was revised based on this study. In *Pseudomonas fluorescens *PF0-1, Kim et al. searched unrecognized genes with cell fractionation data (global, soluble, and insoluble) followed by off-line two dimensional liquid chromatography combined with tandem mass spectrometry analysis [8]. They found 16 novel genes of which six were intergenic region, nine overlapped with antisense predicted genes, and one overlapped with a predicted gene in another reading flame in the same direction. Payne et al., evaluated the genomes of *Yersinia pestis *with proteomic analysis for complement genome annotation, and 21 other *Yersinia *genomes in public databases were improved, including four new CDSs [4]. One of the excellent adaptations of proteomics to genome annotation was provided for the hyperthermophilic crenarchaeon, *Aeropyrum pernix*. The number of proteins encoded by *A. pernix *has been the matter of some debate because of its high GC content and codon usage [13]. Proteomic analysis of this archaeon provided useful information, including 19 newly identified CDSs [7]. The results of proteomic analysis were used as a reliable index for the development of further gene annotation methods. In *S. pyogenes*, a number of CDSs remain as "(conserved) hypothetical proteins", whereas 13 intra-species genomes were revealed. Despite the strain SF370 being widely used in many researchers, the annotation has remained almost the same as when it was published in the public database. We envisioned that the re-evaluation of the SF370 genome with proteomic experimental evidence would provide useful information.

We identified nine novel genes that were transcribed and translated in SF370, based on assignments from MS/MS spectra from a list of six-frame ORFs rather than a list of known CDSs. Two out of these nine genes were identified in our previously report [27], and the transcriptions of both of these genes were verified by RT-PCR (Figure 1). OppA is believed to be a lipoprotein associated with virulence in mice [36]. The oligopeptide permease complex consists of a periplasmic binding protein (OppA), two transmembrane proteins (OppB and OppC), and two membrane-associated cytoplasmic ATPases (OppD and OppF) on a polycistronic operon [37]. CsrR, also known as CovR, is a unit of a two component signaling system that is associated with stressors, such as temperature, salt concentration, pH, antibiotics, and iron starvation [38-40]. In addition, the CsrR/S system is known to regulate several virulence factors, such as the hyaluronic acid capsule, streptolysin S, streptokinase, and pyrogenic exotoxin B (SpeB) [41]. The CDS in ORF6306 encodes a fibronectin binding protein with a molecular weight of 85.1 kDa, and is believed to be involved in adhesion to the host cell surfaces. Although two other fibronectin binding proteins, SPy0430 and SPy1013, were annotated in SF370, neither of them could be detected in our proteome analysis. ORF5890 contains a CDS that encodes a 96.7 kDa enzyme that is considered to be a bifunctional acetaldehyde-CoA/alcohol dehydrogenase (EC 1.2.1.10 and 1.1.1.1). Four genes encoded by novel ORFs are believed to possess relatively low molecular weights; ORF15403 (26.6 kDa), ORF5890 (22.6 kDa), ORF703 (20.7 kDa), and ORF106976 (11.5 kDa). The full length of ORF106976 is corresponds to 105 amino acid residues. Although the homologous ORF was previously determined in MGAS315, the annotation for ORF106976 in SF370 has been omitted, probably because of its short length.

Unexpectedly, relatively few (nine) genes/novel CDSs were discovered in the SF370 genome, which possesses approximetely100 fewer CDSs compared to other GAS genomes. The number of new CDSs was comparable with previous reports [2,8,13]. In this study, two or more MS/MS spectra matching a unique peptide sequence in an ORF were used as the criterion for protein identification. Although the main goal of this study was a precise re-evaluation of SF370 genomes, this criterion may be too strict for the short length ORFs. The criteria that the identification of a protein was judged by one MS/MS spectrum matching to a unique peptide sequence will be considerable for the screening of unidentified CDS using a six-frame database. Alternatively, we suggest that an analysis that integrates proteomics and tiling DNA arrays should identify more of the short-length unrecognized ORFs. Although it would be easy to find unrecognized genes in a genome by several in silico strategies, such as intra-species genome comparison or searching with GO annotation, further experimental verification by the presence of mRNA or proteins encoded the genes is important. Proteomics-driven re-annotation with a six-frame database allows the identification of unrecognized genes with verification of the gene products at the same time.

The other aim of this study was to experimentally characterize hypothetical genes in GAS and to re-annotate hypothetical proteins by comprehensive analysis. Transcriptomic and/or proteomic analysis to generate functional annotations for hypothetical genes has been widely applied to many living organisms [9-12]. This assignment generated functional annotations for 54 CDSs (9.71% of HyPs) in *Desulfovibrio vulgaris*, 538 CDSs (33.1% of HyPs) in *Shewanella oneidensis*, and 129 (10.6% of HyPs) in the *Haemophilus influenza *genome [9-11]. In the SF370 genome, approximately 40% of proteins had been annotated as "hypothetical" or "conserved hypothetical" proteins. We identified 126 hypothetical proteins in three cellular fractions under three different culture conditions. Proteomics-driven functional annotation can help to not only deduce the response of cells under stressful culture conditions, as in transcriptome analysis, but can also be used to deduce the cellular location of protein expression [10]. The absolute quantification of proteins should establish the number of peptide sequences that are detected under each culture condition, and whether the cellular fractions reflect the abundance of a particular protein [42,43]. Furthermore, the homology search-based annotation, including GO, SignalP, and SOSUI, were integrated into proteomic experimental evidence of the annotation for unrecognized proteins. This integrated functional annotation provided interesting information for unknown proteins. For example, SPy0843 was assigned to the "cell" GO term and had a SignalP score 0.898. This protein was only identified from the insoluble fraction, and was expressed at a relatively high abundance in the static and CO_2 _culture conditions rather than under shaking conditions, by the proteomic analysis. It is speculated that the product of SPy0843 may be located in the cell membrane or cell wall, may be associated with the Sec pathway, and be upregulated under non-shaking culture conditions. Another example is SPy0317, which was assigned the GO terms "cell envelope", "external encapsulating structure", "transport", and "transporter activity", was estimated to have one membrane spanning domain by SOSUI, and had a SignalP score 0.999. The product of SPy0317 was universally observed in all cellular fractions, and was relatively highly expressed under shaking culture conditions. It is speculated that SPy0317 is secreted via the Sec pathway and is involved in transport of substances, especially under shaking culture conditions, which mimics mechanical or oxygenic stress. Other interesting examples were SPy1260 and SPy1262, which were identified with relatively high numbers of MS/MS spectra, despite both of them being assigned no GO terms. They should merit further biochemical and biological investigation.

A high degree of protein variation was observed in the supernatant compared to the insoluble and soluble fractions of the cell (Figure 2). Our previous reports suggested that stressors, such as addition of antibiotics [39,44], influenced the expressions of extracellular proteins. These results suggest that GAS cells change their expression patterns of extracellular proteins when adapting to environmental stresses. In contrast to extracellular proteins, core proteins were easily identified in cell-body fractions under the different culture conditions. It is hypothesized that the protein components that we observed were a consequence of growth during the stationary phase of the cultures. For example, a previous report indicated that the effect of different culture atmospheres modulated surface structures. Bisno et al. reported that the expression level of the M protein of the cell wall-associated fraction was greater in 5% CO_2 _culture conditions [45]. Our results also confirmed this hypothesis (Additional file 4). Interestingly, the highest amounts of M protein in the supernatant were observed under shaking culture conditions. We speculate that the M protein is detached from the cell wall because of the mechanical effects of shaking, although this should be investigated further.

## Conclusions

The proteome of *S. pyogenes *SF370 was characterized by shotgun LC-MS/MS with a non-biased, six-frame translation of open reading frames of the actual genome sequence. In this study, nine proteins were discovered as novel ORFs in SF370, with the validation of their corresponding mRNAs. Furthermore, functional annotation was obtained for 126 hypothetical proteins (22.2% out of all hypothetical proteins). To elucidate the dynamic responses of GAS cells to the environment requires more extensive analysis, which can compare proteomic profiles for different culture conditions, such as atmospheric compositions, culture media, growth phases, temperature, mechanical stress, and the addition of antibiotics. Although effort has been made to illustrate the proteomic profiles of *S. pyogenes*, several proteins may be inadequately evaluated because of unrecognized CDSs in genomes, or the absence of well-characterized annotations, such as for HyPs and CHyPs. Notably, the selection of a reliable database, such as six-frame amino acid sequences derived from actual genome DNA sequences, should be used to ensure reliable proteomic analysis.

The re-evaluation of a genome by proteomic evidence is useful; however, not all the proteins could be identified in a series of experiments because they may not all be expressed at the same time, or because of technical problems. The integrated (re-)evaluation of genomes with the proteomic and transcriptomic analysis, and similarity-based bioinformatics analysis could provide more reliable and useful annotations.

## Methods

### *In silico *Genome Analysis

We studied the genome sequences of *S. pyogenes *in the NCBI database to obtain the length of total chromosomal DNA and the length and number of CDSs, including functional RNAs (rRNA and tRNA), protein coding genes, and others. CDS coverage was evaluated using the total length of CDSs. Accession numbers, genome submission years, and related reference articles for each genome are listed in Additional file 1.

### Bacterial Growth Conditions

*S. pyogenes *SF370 was obtained from the genome-sequencing program at the University of Oklahoma's Advanced Center for Genome Technology [17]. SF370 was cultured at 37°C in 25 mL of brain-heart infusion broth (Eiken, Tokyo, Japan), supplemented with 0.3% yeast extract (Becton Dickinson, Franklin Lakes, NJ) without shaking (static conditions), with shaking at 180 rpm (shaking conditions), or under 5% CO_2 _without shaking (CO_2 _conditions).

### Shotgun Proteomic Analysis

Bacteria were cultured for 14 h under each condition and harvested by centrifugation at 14,000 × g for 10 min. The supernatant was used as the supernatant fraction. Bacterial cells were re-suspended in 10 mL of PBS and then disrupted using a French press. After centrifugation at 14,000 × g for 10 min, supernatant was recovered as the soluble fraction, and the resulting pellet was re-suspended in PBS as the insoluble fraction. Both supernatant and soluble fractions were further concentrated with trichloroacetic acid-acetone, as described previously [44]. Each protein mixture was then digested in solution with a phase transfer surfactant [46]. In brief, a protein mixture was dissolved in 100 μL of solution buffer containing 50 mM ammonium bicarbonate, 8 M urea, and 1% (w/w) sodium deoxycholate. The crude protein solution (100 μL) was incubated with 100 mM dithiothreitol for 30 min at 60°C. Iodoacetamide (final concentration 100 mM) was then added and incubated for 30 min at room temperature in the dark. After incubation, 1 μg of Lysyl Endopeptidase (Wako Pure Chemical Industries, Ltd., Osaka, Japan) was added and incubation continued for 1 hour at 37°C. The sample solution was diluted four-fold with ultrapure water, after which 1 μg of Trypsin Gold, Mass Spectrometry Grade (Promega Co., MI) was added into the solution and incubation continued for 1 h at 37°C. An equal volume of ethyl acetate was added to the solution, and the mixture was acidified with trifluoroacetic acid (final concentration 0.5% v/v). The solution was mixed and centrifuged at 14,000 × g for 2 min, and the aqueous phase was collected. The generated peptide mixture was loaded onto the LC-MS/MS instrument. Shotgun proteomic analysis was performed using an LTQ-Orbitrap XL mass spectrometer (Thermo Fisher Scientific Inc., San Jose, CA) combined with a Paradigm MS4 LC system (Michrom BioResources, Inc., Auburn, CA), equipped with a 75 μm i.d. capillary LC column using 45 min LC separations. Full MS spectra (400-2,000 *m/z*, resolution of 100,000 each) were obtained with Orbitrap XL and product ion spectra were obtained with top 7 data-dependent MS/MS scan of LTQ.

### Protein Identification and Database Construction

The product ion mass lists were generated with the program extract_msn provided by the manufacturer (Thermo Fisher Scientific Inc.), and subjected to the program MASCOT (Matrix Science Inc., Boston, MA) along with in-house amino acid sequence database sets. The search parameters were the following: one missed cleavage permitted, variable modifications were considered for oxidation in methionine, phosphorylation in serine, threonine, and tyrosine, mass tolerance for precursor ions was ± 10 ppm, mass tolerance for fragment ions was ± 0.8 Da, the threshold for peptide identification was 0.05.

For the screening of novel CDSs, a six-frame amino acid database was constructed from the genome DNA sequence of SF370. In the case of a gene that was designated as a pseudogene due to truncation by frameshift from point mutations, insertions or deletions, or a gene that overlapped another reading frame gene, the requirement of an ATG start methionine and the limitation of ORF length were dispensable.

For the identification and re-evaluation of HyPs, an amino acid sequence database, which consisted of 1,697 coding sequences in the genome analysis supplemented by nine novel proteins identified in this study (described in the Results) was used. Proteins with more than two unique peptide sequences among the ORFs were identified. Shotgun proteomic analysis was performed in triplicate for each condition: supernatant, soluble fraction, and insoluble fraction. The proteomic data were converted to PRIDE xml format with PRIDE converter (ver. 2.5.3) and deposited on PRIDE database (http://www.ebi.ac.uk/pride/), with accession number 19230 for six-flame database and 19231 for in-house amino acid database, respectively [47].

### Reverse Transcription PCR

Bacteria were cultured for 5 h under each condition and total RNA was extracted and purified with an RNeasy^® ^Mini kit (QIAGEN, Hilden, Germany). Trace DNA in the RNA preparation was removed with TURBO DNA-*free *treatment (Ambion Inc., Austin, TX). For RT-PCR, RNA was reverse transcribed with Superscript II™ Reverse Transcriptase (Invitrogen, Carlsbad, CA) in a 50 μL volume according to the manufacturer's recommendations. One microliter of cDNA was used as a template for RT-PCR with each specific primer pair. DNA contamination was confirmed by performing mock-RT-PCR without reverse transcriptase. The primer pairs and cycle numbers for PCR tests are listed in Additional file 7. Other PCR profiles, including an annealing temperature of 55°C, and an extension temperature of 72°C for 30 seconds, were commonly used for all primer pair sets.

### Bioinformatics and Statistical Analyses

The GAS genome information was processed using the Artemis (Release 11) program [48]. The deduced amino acid sequences of GAS genes were compared using the ClustalX program (ver. 2.0.9) [49]. The presence of signal peptide sequences was analyzed using the SignalP 3.0 Server (http://www.cbs.dtu.dk/services/SignalP/) [29,30]. Membrane spanning domains were estimated using the SOSUI program (http://bp.nuap.nagoya-u.ac.jp/sosui/) [28]. The Gene Ontology terms were assigned to unrecognized CDSs and hypothetical proteins using the Blast2GO suite [50,51].

## List of Abbreviations

CDS: Coding Sequence; CHyP: Conserved hypothetical protein; FDR: False discovery rate; GAS: Group A streptococci; HyP: Hypothetical protein; LC-MS/MS: Liquid chromatography combined with tandem mass spectrometry; NCBI: National Center of Biotechnology Information; RT-PCR: Reverse transcriptional PCR

## Competing interests

The authors declare that they have no competing interests.

## Authors' contributions

AO carried out the main component of this study. KY helped to draft the manuscript. Both authors read and approved the final manuscript.

## Authors' information

AO: Ph. D., Assistant Professor of Molecular Bacteriology department, Nagoya University Graduate School of Medicine.

KY: Ph. D., Assistant Professor of Molecular Bacteriology department, Nagoya University Graduate School of Medicine.

## Supplementary Material

Additional file 1 **Cross-sectional Genome Overview of GAS**. Thirteen chromosomal DNA sequences were obtained from the NCBI database. CDS length and coverage, number of genes, number of protein coding genes, and average lengths of protein coding genes were calculated from the information for each genome. The CDS region indicates the total length of genes annotated in each genome. Number of genes refers to those counted as tagged as "gene" in a particular genome. The genes that are annotated as protein coding regions are the number of protein coding genes. The genome overview is listed for the genome submitted or updated year. a) The gene predictor used in this strain was not clearly stated in the manuscript, but estimated via citation. b) The CDS coverage and the number of genes in Manfredo were not analyzed (NA) because of an annotation format that differed from other genomes. Click here for file

Additional file 2 **Overview of the shotgun proteomic analysis**. Using 3 different culture conditions (static; without shaking, CO_2_; under 5% CO_2 _condition without shaking, and shake; with shaking), GAS SF370 tryptic-digested peptide was analyzed with LC-MS/MS. Approximately 7,000 spectra were queried with MASCOT server with a real and randomized decoy database for each six-frame and refined amino acid database (read DB) consisting of 1,707 CDSs. The identification certainty was evaluated by the false discovery rate (FDR). Click here for file

Additional file 3 **Candidate CDS found in this study**. The ORFs that were assigned to more than two unique sequences are listed in this table with Gene Ontology annotation. Total numbers of average identified unique sequences of each experiment group are listed. mRNA encoding CDS candidates was amplified with RT-PCR (+) or not (-). Abbreviations: ORF ID, unique number of ORF in the six frame database in this study; Mw and pI, molecular weight and isoelectric point deduced from the amino acid sequence; SNT, supernatant fraction; SOL, soluble fraction; INS, insoluble fraction. n/a; not available. Click here for file

Additional file 4 **Table of identified proteins with in-house refined database**. Abbreviations; a) Synonym, Tag number in SF370 genome; b) Gene, gene name; c) PID, GI number of protein in NCBInr database; d) COGs code, abbreviation of functional categories in Clusters of Orthologous Groups project. Each one letter abbreviation is detailed in the manuscript, and Additional file 5 and 6; e) MSD, the number of membrane spanning domain that calculated by SOSUI program; f) SP, the probability score of the signal peptide prediction with SignalP 3.0 program (Hidden Markov Model); g) Abbreviation in "static", "CO_2_", and "shake" columns: score, MASCOT score; %AA, coverage percent in amino acid; seq, spectrum matched number for unique sequence; emPAI, experimental modified Peptide Abundant Index. Click here for file

Additional file 5 **Annotations for "Conserved hypothetical proteins"**. "Conserved hypothetical proteins", which were assigned more than two unique sequences, are listed in this table with homology search based annotation, such as Gene Ontology. Total numbers of average identified unique sequences in each experiment group are listed. Abbreviations in the description column; Synonym, tag number in the SF370 genome; a) Abbreviations in the "location" column; S, secreted protein (supernatant fraction); C, cytoplasmic protein (soluble fraction); W, cell wall associated protein (insoluble fraction), uni; universally identified in all cellular fractions; the number indicates average of MS/MS spectrum number that was assigned to unique peptide sequences. b) Abbreviations in the "condition" column; sta, culture under static growth conditions; co, culture under 5% CO_2 _culture conditions; sha, culture under shaking conditions; uni, universally identified in all three culture conditions. The number indicates average of MS/MS spectrum number that was assigned to unique peptide sequences. c) COGs, abbreviation of functional categories in Clusters of Orthologous Groups project. "D", Cell cycle control, cell division, chromosome partitioning; "E", Amino acid transport and metabolism; "G", Carbohydrate transport and metabolism; "H", Coenzyme transport and metabolism; "I", Lipid transport and metabolism; "J", Translation, ribosomal structure and biogenesis; "K", Transcription; "M", Cell wall/membrane/envelope biogenesis; "O", Posttranslational modification, protein turnover, chaperones; "P", Inorganic ion transport and metabolism; "Q", Secondary metabolites biosynthesis, transport and catabolism; "R", General function prediction only; "S", Function unknown; "T", Signal transduction mechanisms; "U", Intracellular trafficking, secretion, and vesicular transport; "V", Defense mechanisms; and "-", Not classified into COGs; d) MSD, the number of membrane spanning domain calculated by the SOSUI program, in Reference 48. e) SP, the probability score of signal peptide prediction with the SignalP 3.0 program (Hidden Markov Model), in Reference 29, 30 Click here for file

Additional file 6 **Annotations for "Hypothetical proteins"**. "Hypothetical proteins", which were assigned more than two unique sequences, are listed in this table with homology search based annotation, such as Gene Ontology. Total numbers of average identified unique sequences in each experiment group are listed. Abbreviations in the description column; Synonym, tag number in the SF370 genome; a) Abbreviations in the "location" column; S, secreted protein (supernatant fraction); C, cytoplasmic protein (soluble fraction); W, cell wall associated protein (insoluble fraction), uni; universally identified in all cellular fractions; the number indicates average of MS/MS spectrum number that was assigned to unique peptide sequences. b) Abbreviations in the "condition" column; sta, culture under static growth conditions; co, culture under 5% CO_2 _culture conditions; sha, culture under shaking conditions; uni, universally identified in all three culture conditions. The number indicates average of MS/MS spectrum number that was assigned to unique peptide sequences. c) COGs, abbreviation of functional categories in Clusters of Orthologous Groups project. "D", Cell cycle control, cell division, chromosome partitioning; "E", Amino acid transport and metabolism; "G", Carbohydrate transport and metabolism; "H", Coenzyme transport and metabolism; "I", Lipid transport and metabolism; "J", Translation, ribosomal structure and biogenesis; "K", Transcription; "M", Cell wall/membrane/envelope biogenesis; "O", Posttranslational modification, protein turnover, chaperones; "P", Inorganic ion transport and metabolism; "Q", Secondary metabolites biosynthesis, transport and catabolism; "R", General function prediction only; "S", Function unknown; "T", Signal transduction mechanisms; "U", Intracellular trafficking, secretion, and vesicular transport; "V", Defense mechanisms; and "-", Not classified into COGs; d) MSD, the number of membrane spanning domain calculated by the SOSUI program, in Reference 48. e) SP, the probability score of signal peptide prediction with the SignalP 3.0 program (Hidden Markov Model), in Reference 29, 30 Click here for file

Additional file 7 **Table listing the information on primers used for RT-PCR assay**. The RT-PCR procedure is detailed in the Methods section. The sequences of each primer, cycle numbers for amplification, and estimated product sizes are listed. Click here for file

## References

[B1] Sivashankari S Shanmughavel P Functional annotation of hypothetical proteins - A review Bioinformation 2006 1 8 335 338 17597916 10.6026/97320630001335 PMC1891709

[B2] Dandekar T Huynen M Regula JT Ueberle B Zimmermann CU Andrade MA Doerks T Sánchez-Pulido L Snel B Suyama M Yuan YP Herrmann R Bork P Re-annotating the *Mycoplasma pneumoniae *genome sequence: adding value, function and reading frames Nucleic acids research 2000 28 17 3278 3288 10.1093/nar/28.17.3278 10954595 PMC110705

[B3] Luo C Hu GQ Zhu H Genome reannotation of *Escherichia coli *CFT073 with new insights into virulence BMC genomics 2009 10 552 10.1186/1471-2164-10-552 19930606 PMC2785843

[B4] Payne SH Huang ST Pieper R A proteogenomic update to *Yersinia*: enhancing genome annotation BMC genomics 11 460 10.1186/1471-2164-11-460 PMC3091656 20687929

[B5] Bocs S Danchin A Medigue C Re-annotation of genome microbial coding-sequences: finding new genes and inaccurately annotated genes BMC bioinformatics 2002 3 5 10.1186/1471-2105-3-5 11879526 PMC77393

[B6] Guo FB Wang J Zhang CT Gene recognition based on nucleotide distribution of ORFs in a hyper-thermophilic crenarchaeon, *Aeropyrum pernix *K1 DNA Res 2004 11 6 361 370 10.1093/dnares/11.6.361 15871459

[B7] Guo FB Lin Y Identify protein-coding genes in the genomes of *Aeropyrum pernix *K1 and Chlorobium tepidum TLS Journal of biomolecular structure & dynamics 2009 26 4 413 420 19108580 10.1080/07391102.2009.10507256

[B8] Kim W Silby MW Purvine SO Nicoll JS Hixson KK Monroe M Nicora CD Lipton MS Levy SB Proteomic detection of non-annotated protein-coding genes in *Pseudomonas fluorescens *Pf0-1 PloS one 2009 4 12 e8455 10.1371/journal.pone.0008455 20041161 PMC2794547

[B9] Kolker E Makarova KS Shabalina S Picone AF Purvine S Holzman T Cherny T Armbruster D Munson RS Jr Kolesov G Frishman D Galperin MY Identification and functional analysis of 'hypothetical' genes expressed in *Haemophilus influenzae* Nucleic acids research 2004 32 8 2353 2361 10.1093/nar/gkh555 15121896 PMC419445

[B10] Zhang W Culley DE Gritsenko MA Moore RJ Nie L Scholten JC Petritis K Strittmatter EF Camp DG Smith RD Brockman FJ LC-MS/MS based proteomic analysis and functional inference of hypothetical proteins in *Desulfovibrio vulgaris* Biochemical and biophysical research communications 2006 349 4 1412 1419 10.1016/j.bbrc.2006.09.019 16982031

[B11] Kolker E Picone AF Galperin MY Romine MF Higdon R Makarova KS Kolker N Anderson GA Qiu X Auberry KJ Babnigg G Beliaev AS Edlefsen P Elias DA Gorby YA Holzman T Klappenbach JA Konstantinidis KT Land ML Lipton MS McCue LA Monroe M Pasa-Tolic L Pinchuk G Purvine S Serres MH Tsapin S Zakrajsek BA Zhu W Zhou J Larimer FW Lawrence CE Riley M Collart FR Yates JR Smith RD Giometti CS Nealson KH Fredrickson JK Tiedje JM Global profiling of *Shewanella oneidensis *MR-1: expression of hypothetical genes and improved functional annotations Proceedings of the National Academy of Sciences of the United States of America 2005 102 6 2099 2104 10.1073/pnas.0409111102 15684069 PMC548307

[B12] Jiang SY Christoffels A Ramamoorthy R Ramachandran S Expansion mechanisms and functional annotations of hypothetical genes in the rice genome Plant physiology 2009 150 4 1997 2008 10.1104/pp.109.139402 19535473 PMC2719134

[B13] Yamazaki S Yamazaki J Nishijima K Otsuka R Mise M Ishikawa H Sasaki K Tago S Isono K Proteome analysis of an aerobic hyperthermophilic crenarchaeon, *Aeropyrum pernix *K1 Mol Cell Proteomics 2006 5 5 811 823 10.1074/mcp.M500312-MCP200 16455681

[B14] Chaussee MA McDowell EJ Chaussee MS Proteomic analysis of proteins secreted by *Streptococcus pyogenes* Methods in molecular biology (Clifton, NJ 2008 431 15 24 10.1007/978-1-60327-032-8_2 18287744

[B15] Severin A Nickbarg E Wooters J Quazi SA Matsuka YV Murphy E Moutsatsos IK Zagursky RJ Olmsted SB Proteomic analysis and identification of *Streptococcus pyogenes *surface-associated proteins Journal of bacteriology 2007 189 5 1514 1522 10.1128/JB.01132-06 17142387 PMC1855729

[B16] Chaussee MA Dmitriev AV Callegari EA Chaussee MS Growth phase-associated changes in the transcriptome and proteome of *Streptococcus pyogenes* Archives of microbiology 2008 189 1 27 41 17665172 10.1007/s00203-007-0290-1

[B17] Ferretti JJ McShan WM Ajdic D Savic DJ Savic G Lyon K Primeaux C Sezate S Suvorov AN Kenton S Lai HS Lin SP Qian Y Jia HG Najar FZ Ren Q Zhu H Song L White J Yuan X Clifton SW Roe BA McLaughlin R Complete genome sequence of an M1 strain of *Streptococcus pyogenes* Proceedings of the National Academy of Sciences of the United States of America 2001 98 8 4658 4663 10.1073/pnas.071559398 11296296 PMC31890

[B18] Smoot JC Barbian KD Van Gompel JJ Smoot LM Chaussee MS Sylva GL Sturdevant DE Ricklefs SM Porcella SF Parkins LD Beres SB Campbell DS Smith TM Zhang Q Kapur V Daly JA Veasy LG Musser JM Genome sequence and comparative microarray analysis of serotype M18 group A *Streptococcus *strains associated with acute rheumatic fever outbreaks Proceedings of the National Academy of Sciences of the United States of America 2002 99 7 4668 4673 10.1073/pnas.062526099 11917108 PMC123705

[B19] Beres SB Sylva GL Barbian KD Lei B Hoff JS Mammarella ND Liu MY Smoot JC Porcella SF Parkins LD Campbell DS Smith TM McCormick JK Leung DY Schlievert PM Musser JM Genome sequence of a serotype M3 strain of group A *Streptococcus*: phage-encoded toxins, the high-virulence phenotype, and clone emergence Proceedings of the National Academy of Sciences of the United States of America 2002 99 15 10078 10083 10.1073/pnas.152298499 12122206 PMC126627

[B20] Nakagawa I Kurokawa K Yamashita A Nakata M Tomiyasu Y Okahashi N Kawabata S Yamazaki K Shiba T Yasunaga T Hayashi H Hattori M Hamada S Genome sequence of an M3 strain of *Streptococcus pyogenes *reveals a large-scale genomic rearrangement in invasive strains and new insights into phage evolution Genome research 2003 13 6A 1042 1055 12799345 10.1101/gr.1096703 PMC403657

[B21] Green NM Zhang S Porcella SF Nagiec MJ Barbian KD Beres SB LeFebvre RB Musser JM Genome sequence of a serotype M28 strain of group A *Streptococcus*: potential new insights into puerperal sepsis and bacterial disease specificity The Journal of infectious diseases 2005 192 5 760 770 10.1086/430618 16088825

[B22] Beres SB Richter EW Nagiec MJ Sumby P Porcella SF DeLeo FR Musser JM Molecular genetic anatomy of inter- and intraserotype variation in the human bacterial pathogen group A *Streptococcus* Proceedings of the National Academy of Sciences of the United States of America 2006 103 18 7059 7064 10.1073/pnas.0510279103 16636287 PMC1459018

[B23] Banks DJ Porcella SF Barbian KD Beres SB Philips LE Voyich JM DeLeo FR Martin JM Somerville GA Musser JM Progress toward characterization of the group A *Streptococcus *metagenome: complete genome sequence of a macrolide-resistant serotype M6 strain The Journal of infectious diseases 2004 190 4 727 738 10.1086/422697 15272401

[B24] Holden MT Scott A Cherevach I Chillingworth T Churcher C Cronin A Dowd L Feltwell T Hamlin N Holroyd S Jagels K Moule S Mungall K Quail MA Price C Rabbinowitsch E Sharp S Skelton J Whitehead S Barrell BG Kehoe M Parkhill J Complete genome of acute rheumatic fever-associated serotype M5 *Streptococcus pyogenes *strain manfredo Journal of bacteriology 2007 189 4 1473 1477 10.1128/JB.01227-06 17012393 PMC1797351

[B25] McShan WM Ferretti JJ Karasawa T Suvorov AN Lin S Qin B Jia H Kenton S Najar F Wu H Scott J Roe BA Savic DJ Genome sequence of a nephritogenic and highly transformable M49 strain of *Streptococcus pyogenes* Journal of bacteriology 2008 190 23 7773 7785 10.1128/JB.00672-08 18820018 PMC2583620

[B26] Sumby P Porcella SF Madrigal AG Barbian KD Virtaneva K Ricklefs SM Sturdevant DE Graham MR Vuopio-Varkila J Hoe NP Musser JM Evolutionary origin and emergence of a highly successful clone of serotype M1 group A *Streptococcus *involved multiple horizontal gene transfer events The Journal of infectious diseases 2005 192 5 771 782 10.1086/432514 16088826

[B27] Okamoto A Hasegawa T Yamada K Ohta M Application of both high-performance liquid chromatography combined with tandem mass spectrometry shotgun and 2-D polyacrylamide gel electrophoresis for streptococcal exoproteins gave reliable proteomic data Microbiology and immunology 2011 55 2 84 94 10.1111/j.1348-0421.2010.00302.x 21204954

[B28] Mitaku S Hirokawa T Tsuji T Amphiphilicity index of polar amino acids as an aid in the characterization of amino acid preference at membrane-water interfaces Bioinformatics (Oxford, England) 2002 18 4 608 616 10.1093/bioinformatics/18.4.608 12016058

[B29] Bendtsen JD Nielsen H von Heijne G Brunak S Improved prediction of signal peptides: SignalP 3.0 Journal of molecular biology 2004 340 4 783 795 10.1016/j.jmb.2004.05.028 15223320

[B30] Nielsen H Engelbrecht J Brunak S von Heijne G Identification of prokaryotic and eukaryotic signal peptides and prediction of their cleavage sites Protein engineering 1997 10 1 1 6 10.1093/protein/10.1.1 9051728

[B31] Canchaya C Desiere F McShan WM Ferretti JJ Parkhill J Brussow H Genome analysis of an inducible prophage and prophage remnants integrated in the *Streptococcus pyogenes *strain SF370 Virology 2002 302 2 245 258 10.1006/viro.2002.1570 12441069

[B32] Overbeek R Larsen N Walunas T D'Souza M Pusch G Selkov E Jr Liolios K Joukov V Kaznadzey D Anderson I Bhattacharyya A Burd H Gardner W Hanke P Kapatral V Mikhailova N Vasieva O Osterman A Vonstein V Fonstein M Ivanova N Kyrpides N The ERGO genome analysis and discovery system Nucleic acids research 2003 31 1 164 171 10.1093/nar/gkg148 12519973 PMC165577

[B33] Frishman D Mironov A Mewes HW Gelfand M Combining diverse evidence for gene recognition in completely sequenced bacterial genomes Nucleic acids research 1998 26 12 2941 2947 10.1093/nar/26.12.2941 9611239 PMC147632

[B34] Salzberg SL Delcher AL Kasif S White O Microbial gene identification using interpolated Markov models Nucleic acids research 1998 26 2 544 548 10.1093/nar/26.2.544 9421513 PMC147303

[B35] Van Domselaar GH Stothard P Shrivastava S Cruz JA Guo A Dong X Lu P Szafron D Greiner R Wishart DS BASys: a web server for automated bacterial genome annotation Nucleic acids research 2005 33 Web Server W455 459 10.1093/nar/gki593 PMC1160269 15980511

[B36] Wang CH Lin CY Luo YH Tsai PJ Lin YS Lin MT Chuang WJ Liu CC Wu JJ Effects of oligopeptide permease in group A Streptococcal infection Infection and immunity 2005 73 5 2881 2890 10.1128/IAI.73.5.2881-2890.2005 15845494 PMC1087318

[B37] Podbielski A Pohl B Woischnik M Korner C Schmidt KH Rozdzinski E Leonard BA Molecular characterization of group A streptococcal (GAS) oligopeptide permease (opp) and its effect on cysteine protease production Molecular microbiology 1996 21 5 1087 1099 10.1046/j.1365-2958.1996.661421.x 8885277

[B38] Dalton TL Scott JR CovS inactivates CovR and is required for growth under conditions of general stress in *Streptococcus pyogenes* Journal of bacteriology 2004 186 12 3928 3937 10.1128/JB.186.12.3928-3937.2004 15175307 PMC419969

[B39] Sawai J Hasegawa T Kamimura T Okamoto A Ohmori D Nosaka N Yamada K Torii K Ohta M Growth phase-dependent effect of clindamycin on production of exoproteins by *Streptococcus pyogenes* Antimicrobial agents and chemotherapy 2007 51 2 461 467 10.1128/AAC.00539-06 17101685 PMC1797754

[B40] Froehlich BJ Bates C Scott JR *Streptococcus pyogenes *CovRS mediates growth in iron starvation and in the presence of the human cationic antimicrobial peptide LL-37 Journal of bacteriology 2009 191 2 673 677 10.1128/JB.01256-08 18996992 PMC2620807

[B41] Heath A DiRita VJ Barg NL Engleberg NC A two-component regulatory system, CsrR-CsrS, represses expression of three *Streptococcus pyogenes *virulence factors, hyaluronic acid capsule, streptolysin S, and pyrogenic exotoxin B Infection and immunity 1999 67 10 5298 5305 10496909 10.1128/iai.67.10.5298-5305.1999 PMC96884

[B42] Shinoda K Tomita M Ishihama Y emPAI Calc--for the estimation of protein abundance from large-scale identification data by liquid chromatography-tandem mass spectrometry Bioinformatics (Oxford, England) 2010 26 4 576 577 10.1093/bioinformatics/btp700 20031975

[B43] Ishihama Y Schmidt T Rappsilber J Mann M Hartl FU Kerner MJ Frishman D Protein abundance profiling of the *Escherichia coli *cytosol BMC genomics 2008 9 102 10.1186/1471-2164-9-102 18304323 PMC2292177

[B44] Tanaka M Hasegawa T Okamoto A Torii K Ohta M Effect of antibiotics on group A *Streptococcus *exoprotein production analyzed by two-dimensional gel electrophoresis Antimicrobial agents and chemotherapy 2005 49 1 88 96 10.1128/AAC.49.1.88-96.2005 15616280 PMC538853

[B45] Bisno AL Brito MO Collins CM Molecular basis of group A streptococcal virulence The Lancet infectious diseases 2003 3 4 191 200 10.1016/S1473-3099(03)00576-0 12679262

[B46] Masuda T Saito N Tomita M Ishihama Y Unbiased quantitation of *Escherichia coli *membrane proteome using phase transfer surfactants Mol Cell Proteomics 2009 8 12 2770 2777 10.1074/mcp.M900240-MCP200 19767571 PMC2816013

[B47] Barsnes H Vizcaino JA Eidhammer I Martens L PRIDE Converter: making proteomics data-sharing easy Nature biotechnology 2009 27 7 598 599 10.1038/nbt0709-598 19587657

[B48] Rutherford K Parkhill J Crook J Horsnell T Rice P Rajandream MA Barrell B Artemis: sequence visualization and annotation Bioinformatics (Oxford, England) 2000 16 10 944 945 10.1093/bioinformatics/16.10.944 11120685

[B49] Larkin MA Blackshields G Brown NP Chenna R McGettigan PA McWilliam H Valentin F Wallace IM Wilm A Lopez R Clustal W and Clustal X version 2.0 Bioinformatics (Oxford, England) 2007 23 21 2947 2948 10.1093/bioinformatics/btm404 17846036

[B50] Ashburner M Ball CA Blake JA Botstein D Butler H Cherry JM Davis AP Dolinski K Dwight SS Eppig JT Gene ontology: tool for the unification of biology. The Gene Ontology Consortium Nature genetics 2000 25 1 25 29 10.1038/75556 10802651 PMC3037419

[B51] Gotz S Garcia-Gomez JM Terol J Williams TD Nagaraj SH Nueda MJ Robles M Talon M Dopazo J Conesa A High-throughput functional annotation and data mining with the Blast2GO suite Nucleic acids research 2008 36 10 3420 3435 10.1093/nar/gkn176 18445632 PMC2425479

